# A Narrative Review of Current and Investigational Therapies in Hypertrophic Cardiomyopathy

**DOI:** 10.3390/biomedicines13061327

**Published:** 2025-05-29

**Authors:** Ian Ogurek, Randeep Gill, Vasiliki Tasouli-Drakou, Ronnie Joseph, Arbab Khalid, Nazanin Houshmand, Tahir Tak

**Affiliations:** 1 Department of Internal Medicine, Kirk Kerkorian School of Medicine, University of Nevada, Las Vegas, NV 89106, USA; randeep.gill@unlv.edu (R.G.); vasiliki.tasoulidrakou@unlv.edu (V.T.-D.); josepr4@unlv.nevada.edu (R.J.); arbab.khalid@unlv.edu (A.K.); 2 Department of Cardiovascular Medicine, University of Nevada, Las Vegas, NV 89106, USA; nazanin.houshmand@unlv.edu (N.H.); tahir.tak@va.gov (T.T.)

**Keywords:** hypertrophic cardiomyopathy, advanced therapies, procedural interventions

## Abstract

Hypertrophic cardiomyopathy is the most prevalent inherited cardiac condition, distinguished by an autosomal dominant inheritance pattern. Diagnosis can be achieved through echocardiography, cardiac magnetic resonance imaging, and genetic testing. Recently, significant advancements have occurred in pharmacological and invasive therapies that are transforming the management of hypertrophic cardiomyopathy, including the introduction of cardiac myosin inhibitors, radiofrequency ablation, and gene editing. Additional trials are being conducted on investigational therapies, with the results anticipated in the near future. This review aims to provide a concise overview of both currently approved and investigational treatments for hypertrophic cardiomyopathy.

## 1. Introduction

**Figure 1:**
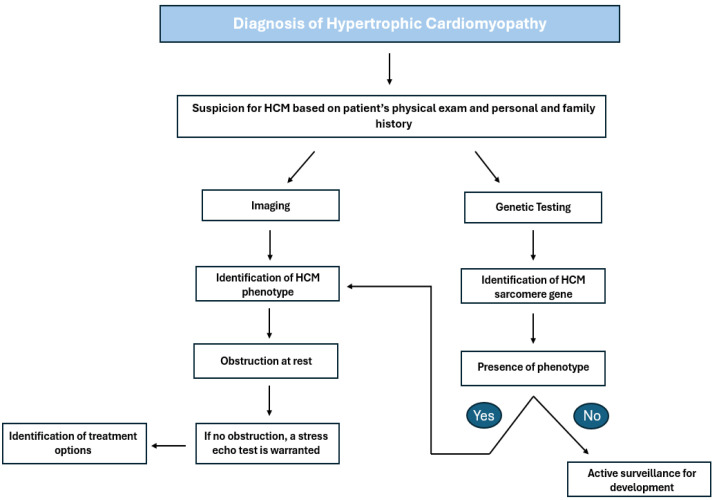
Outflow diagram highlighting the steps for diagnosing and treating HCM based on ACC guidelines. HCM: hypertrophic cardiomyopathy [4].

Hypertrophic cardiomyopathy (HCM) is the most frequently inherited cardiac condition, marked by an autosomal dominant mutation that impacts the genes encoding the cardiac sarcomere proteins responsible for contractile function [1]. HCM can be categorized into two main types: hypertrophic obstructive cardiomyopathy (HOCM), which features left ventricular outflow obstruction due to septal wall hypertrophy, and non-obstructive HCM (nHCM), which does not exhibit a hemodynamically significant outflow gradient [2]. Despite its prevalence of 1 in 500 individuals globally, the clinical significance of HCM has often been overlooked [3]. Since only a small fraction of cases are recognized clinically, advancements in echocardiography and cardiac magnetic resonance (CMR) have improved the diagnosis and management of HCM [4]. These noninvasive imaging techniques enable clinicians to evaluate key cardiac features, such as left ventricular (LV) wall thickness, contraction, and relaxation, as well as identify outflow tract obstruction, contributing to higher diagnosis rates [5]. The diagnostic criteria are concisely outlined in Table 1 and Figure 1, reflecting the 2024 AHA and ACC guidelines for HCM diagnosis. In recent years, there have been significant advancements in novel pharmacological approaches to treating HCM, alongside promising investigational therapies that are reshaping the field. However, the current literature remains limited regarding the latest approved treatments and emerging therapies under investigation. Therefore, this review article aims to offer a thorough overview of both approved and investigational therapies for managing HCM and to provide new insight into the field’s future.

## 2. Methods

This review was designed as a narrative review with the intent to incorporate a comprehensive range of studies, including practice guidelines, randomized controlled trials, systematic reviews, and observational studies, which focus on various approved and investigational therapeutic interventions for the treatment of hypertrophic cardiomyopathy. A literature search was conducted across multiple bibliographic databases, including PubMed, MEDLINE, and Google Scholar. The narrative review utilizing these databases examined contemporary research regarding advancements in therapies within the field of HCM. The keywords employed in this search include “hypertrophic obstructive cardiomyopathy”, “HCM”, “HCM new therapies”, “HCM treatment”, and “HCM guidelines”. This comprehensive review addressed the following questions: “What are current therapies for hypertrophic cardiomyopathy?” and “Are there any recent advances in HCM therapies?” Considering that the most recent AHA guidelines incorporated material published through May 2023, the date range for data collection was adjusted accordingly. The authors also explored clinical trials, systematic reviews, and meta-analyses primarily published after 1 June 2023. Nonetheless, articles outside of this timeframe were also considered. Case reports, case series, and publications available only as abstracts were excluded from the search criteria. All references were screened for relevance, and those not directly examining interventions aimed at hypertrophic cardiomyopathy were likewise excluded.

## 3. Indications for Treatment

Hypertrophic cardiomyopathy (HCM) in isolation does not invariably necessitate treatment. Interventions, whether pharmacologic or invasive, are reserved for individuals diagnosed with HCM who present with symptoms, particularly those identified with obstructive HCM (HOCM). Historically, pharmacologic treatment has been directed toward alleviating symptoms by reducing the left ventricular outflow tract (LVOT) gradient. Nevertheless, significant progress has been achieved in both pharmacological alternatives and invasive techniques, which aim to reduce symptoms and enhance various other metrics, as is delineated below.

## 4. Current Pharmacological and Investigative Therapies

### 4.1. Beta Blockers and Non-Dihydropyridine Calcium Channel Blockers

Harmful inotropic medications, specifically beta-blockers and non-dihydropyridine calcium channel blockers (namely, diltiazem and verapamil), have traditionally constituted the standard treatment for individuals experiencing symptomatic hypertrophic cardiomyopathy (HCM) to alleviate heart failure symptoms. However, they exert minimal influence on disease progression [4]. Both classes of these medications are endorsed with a Class 1 indication in the AHA/ACC guidelines regarding the management of symptomatic hypertrophic obstructive cardiomyopathy (HOCM). Notably, beta-blockers are recognized as a first-line therapy and, should they be poorly tolerated or ineffective, may be substituted with non-dihydropyridine calcium channel blockers. Typically, these medications are considered palliative options, since they can effectively manage symptoms without significantly altering clinical outcomes. Historically, beta-blockers have been employed as the primary agents in patients with outflow obstruction to mitigate outflow gradients and enhance left ventricular filling time; however, robust evidence supporting their efficacy remains limited [4]. Dosages of beta-blockers are escalated until an improvement in symptoms is observed. Diltiazem and verapamil are frequently utilized as alternatives to beta-blocker therapy. The data substantiating the simultaneous use of beta-blockers in conjunction with non-dihydropyridine calcium channel blockers remain ambiguous. Patients who do not respond favorably to first-line therapies may be evaluated for advanced treatment modalities, including cardiac myosin inhibitors or disopyramide, as is elucidated in subsequent sections.

### 4.2. Disopyramide

Disopyramide is classified as a Class 1a antiarrhythmic agent with a specific Class 1 indication, which may be utilized in the treatment of symptomatic hypertrophic cardiomyopathy (HCM) related to left ventricular outflow tract (LVOT) obstruction, either as a monotherapy or in conjunction with other therapeutic agents [7]. This medication is generally designated for symptomatic patients who have not responded adequately to first-line treatments, including beta blockers or non-dihydropyridine calcium channel blockers. A limited body of literature comparatively evaluates the efficacy of disopyramide against beta blockers and calcium channel blockers. Typically, beta blockers and non-dihydropyridine calcium channel blockers are employed initially due to their ease of administration and favorable safety profile, particularly in comparison to disopyramide, which is often initiated during hospitalization for close monitoring owing to its associated risk for proarrhythmic effects, as is discussed further below. The clinical advantages of disopyramide encompass an enhancement in heart failure symptoms, as evidenced by a reduction in heart failure functional class exceeding one class, along with a significant decline in both resting and provocative outflow gradients. A study conducted by Massera et al. indicated that individuals receiving disopyramide demonstrated a decrease in the resting outflow gradient of more than 30% and a reduction in the provocative gradient exceeding 50% [8]. Furthermore, patients treated with disopyramide exhibited a lower likelihood of requiring an escalation in therapeutic intervention for HCM, such as septal ablation or surgical myomectomy. The safety profile of disopyramide is distinguished as excellent, based on both short- and long-term studies, with the rate of all-cause mortality associated with disopyramide being remarkably low. Despite prior studies expressing apprehensions regarding the potential proarrhythmic effects of the drug, ventricular tachyarrhythmias were reported to be as low as 0.2 percent annually, according to Massera et al. [8]. Additional potential adverse reactions may include anti-cholinergic symptoms, which can be exacerbated with pyridostigmine, as well as increased conduction through the AV node, resulting in an elevated risk for the development of atrial fibrillation. In light of the risk associated with AV nodal enhancement, disopyramide should be administered alongside an agent that modulates conduction through the AV node, such as beta blockers, verapamil, or diltiazem. Importantly, left ventricular ejection fraction remains unaffected by disopyramide in contrast to cardiac myosin inhibitors; consequently, patients on disopyramide are not required to undergo frequent echocardiograms, which may represent a considerable barrier for certain patients. Disopyramide is also regarded as a rapid-acting agent, with some symptom relief observable following the initial dose, and it is comparatively less cost-prohibitive than cardiac myosin inhibitors. The 2024 American Heart Association (AHA)/American College of Cardiology (ACC) Guidelines for the Management of HCM advocate for individualized decision making regarding the use of cardiac myosin inhibitors versus disopyramide on a case-by-case basis [8]. The safety profile and efficacy of disopyramide, as substantiated by long-term follow-up studies, strongly reinforce its significant role in managing symptomatic hypertrophic obstructive cardiomyopathy (HOCM).

### 4.3. Cardiac Myosin Inhibitors

Recently, a significant advancement in treatment options has emerged with the introduction of mavacamten, the first selective allosteric inhibitor of cardiac-specific myosin adenosine triphosphatase. This drug reversibly inhibits actin-myosin cross-bridging in cardiac myocytes [9]. Mavacamten is currently FDA-approved for treating symptomatic HOCM in patients with a normal ejection fraction, and it has a Class I recommendation from the 2024 AHA/ACC guidelines for those who do not respond to beta blockers and CCBs. A meta-analysis of three randomized controlled trials (RCTs) assessing mavacamten (EXPLORER-HCM, MAVERICK-HCM, and VALOR-HCM) revealed improvements in peak oxygen consumption (pVO2), significantly higher rates of enhanced NYHA functional class status, and reduced eligibility for septal reduction therapy (SRT) among patients with HOCM [9]. Notably, there was no significant disparity between treatment and placebo groups regarding significant adverse events (SAEs); however, the mavacamten group experienced increased treatment emergency adverse events (TEAEs), the most common being dizziness, fatigue, and palpitations [9]. Despite its promising early outcomes, mavacamten has limitations. A small number of individuals reported a decrease in left ventricular systolic function, resulting in the discontinuation of the drug, which was either resumed at a lower dose or stopped entirely [9]. Additionally, there are currently no long-term trials to validate the safety of mavacamten compared to disopyramide. Similarly, Aficamten, a reversible selective cardiac myosin inhibitor, is under FDA review for new drug approval. It differs from mavacamten due to its wider therapeutic window and half-life, allowing for dose adjustments every two weeks [10]. The recent 2024 SEQUOIA-HCM trial demonstrated that Aficamten led to a significantly higher peak oxygen consumption gradient than placebo. Furthermore, none of the participants with an ejection fraction below 50% experienced treatment interruptions or heart failure decompensation, a limitation noted in mavacamten trials [10]. In a recent phase 2 trial (REDWOOD-HCM), Aficamten was evaluated in patients with nHCM, showing improvements in NYHA functional class by at least one class and alleviating anginal symptoms. Secondary outcomes, including cardiac biomarkers like NT-proBNP levels, also improved [11]. These trial results emphasize Aficamten’s unique pharmacological properties compared to mavacamten. Cardiac myosin inhibitors potentially transform HCM management, but further research is essential to understand their long-term effects and efficacy.

### 4.4. Ranolazine

Ranolazine has been evaluated as an adjuvant treatment for HOCM to alleviate angina and diastolic dysfunction. The RESTYLE-HCM trial involved symptomatic HCM patients, revealing no significant impact on exercise capacity, diastolic function, or NT-proBNP levels compared to placebo [12]. Presently, the AHA/ACC guidelines do not recognize ranolazine as a first-line option for HOCM. However, the European Society of Cardiology (ESC) recommends its use in patients with angina and nHCM without epicardial coronary disease to lessen symptoms, categorized as a Class IIb recommendation [13]. Despite previous negative studies, ranolazine has the potential to theoretically improve HCM and ischemia associated with diastolic dysfunction, providing symptom relief in carefully selected individuals. Future research may be needed to explore ranolazine’s benefits for HCM further.

### 4.5. Sacubitril/Valsartan in oHCM

Sacubitril/valsartan (Entresto) has established itself as a transformative medication for patients diagnosed with heart failure with reduced ejection fraction. Despite this success, sacubitril/valsartan has been explored as a potential therapeutic agent in patients with hypertrophic cardiomyopathy (HCM), although trials have not demonstrated significant benefits. The open-label, blinded, and randomized SILICOFCM trial conducted in non-obstructive HCM indicated no observable impact on left ventricular hypertrophy, exercise capacity, or diastolic function after 16 weeks of sacubitril/valsartan treatment compared to the standard of care [14]. Despite the medication being well tolerated, Entresto was not found to provide a substantial improvement over placebo in enhancing short-term outcomes among patients with preserved-ejection fraction HCM. Consequently, neither the ACC/AHA nor the ESC guidelines for HCM specifically recommend Entresto for patients with obstructive hypertrophic cardiomyopathy (HOCM) and preserved systolic function. Vasodilators are typically contraindicated in cases of obstructive HCM due to their potential to exacerbate the outflow gradient. The 2020 ACC/AHA guidelines for HCM indicate that if a patient with HCM exhibits an ejection fraction below 50%, they should receive standard heart failure therapy, which includes Entresto (classified as a Class I recommendation) [15].

### 4.6. Angiotensin II Receptor Blockers (ARBs) in oHCM

Angiotensin receptor blockers (ARBs) have been thoroughly investigated as a potential disease-modifying therapy for hypertrophic obstructive cardiomyopathy (HOCM) due to their demonstrated anti-hypertrophic and antifibrotic effects observed in preclinical models. Initial pilot studies indicated that ARBs, such as candesartan, may have the potential to reverse left ventricular (LV) hypertrophy and improve diastolic function in patients with non-obstructive hypertrophic cardiomyopathy (nHCM) [16]. However, larger-scale trials conducted in individuals with established HOCM have produced neutral findings. The INHERIT trial—a randomized, placebo-controlled study lasting 12 months involving losartan in adults diagnosed with overt HCM—revealed no statistically significant differences between the losartan and placebo groups in terms of LV mass, maximum wall thickness, fibrosis burden, diastolic function, exercise tolerance, or symptomatic relief [17]. A meta-analysis of randomized controlled trials (RCTs) reported that the reduction in LV mass following ARB therapy, presumably attributed to lowering blood pressure, conferred no appreciable clinical benefit [18]. Current guidelines regarding HCM do not advocate using ARBs as a chronic management therapy for HOCM patients with preserved ejection fraction (EF). In contrast, there remains active interest in the potential application of ARBs as preventive treatment for genotype-positive individuals or in the early stages of HCM. A contemporary Phase 2trial investigating early sarcomeric HCM indicated that valsartan improved cardiac structure and function, suggesting that early intervention may slow disease progression if initiated before the development of advanced hypertrophy [19].

### 4.7. Perhexiline

The primary factor contributing to the symptoms in hypertrophic cardiomyopathy (HCM) is the extent of left ventricular hypertrophy (LVH). Perhexiline is classified as a coronary vasodilator that was initially developed for use as a prophylactic antianginal agent. Traditional pharmacological interventions have sought to mitigate the outflow obstruction attributable to LVH; however, no existing medications have directly targeted the fundamental myocardial energetics. Gene mutations occurring in sarcomeric proteins are responsible for the onset of LVH. Furthermore, it is proposed that there exists a diminished efficiency of energy utilization within the myocardium of individuals diagnosed with HCM, which ultimately contributes to the progression of LVH.

Fatty acids serve as the primary metabolite utilized by the myocardium under normal conditions. Glucose represents an alternative substrate for adenosine triphosphate (ATP) production in the myocardium, requiring reduced oxygen while providing an equivalent energy yield. The energy metabolism of the myocardium may be redirected to favor the utilization of glucose over fatty acids, consequently enhancing energy efficiency and potentially reversing LVH. Perhexiline acts as an inhibitor of carnitine palmitoyltransferase (CPT-1) and is currently under investigation for the treatment of HCM, with the potential to induce a “Randle Shift”, during which fatty acid oxidation is downregulated, prompting the myocardium to prioritize glucose utilization [20]. Perhexiline possesses the potential to emerge as a first-in-class medication specifically aimed at addressing the underlying myocardial metabolism associated with the development of LVH in HCM. The RESOLVE-HCM trial represents a prospective, double-blinded, and placebo-controlled randomized trial that compares the efficacy of perhexiline to a placebo in individuals exhibiting symptomatic HCM.

## 5. Procedural Interventions

Septal reduction therapy (SRT), which includes septal myectomy (SM), alcohol septal ablation (ASA), or radiofrequency ablation (RFA), is recommended for patients who do not respond to first-line medical management. Furthermore, it carries a Class 1 recommendation for these individuals [6]. These invasive procedures are designed to decrease the left ventricular outflow tract (LVOT) gradient, particularly in patients exhibiting hypertrophic obstructive cardiomyopathy (HOCM) symptoms. The decision regarding which procedure to undertake, when indicated, is contingent upon the facility’s capabilities, existing comorbidities, and the patient’s preferences.

### 5.1. Septal Myectomy (SM)

A septal myectomy, classified as a Class I indication, represents an invasive procedure conducted by a cardiothoracic surgeon. The primary objective of this procedure is to excise excessive tissue from the interventricular septum, thereby alleviating the obstruction of the left ventricle. This operation is executed through an open sternotomy, which provides surgical access and simultaneously entails inherent risks and complications associated with invasive intrathoracic surgery. Evidence suggests that septal myectomy yields favorable outcomes in reducing the resting left ventricular outflow tract (LVOT) gradient for symptomatic patients diagnosed with hypertrophic obstructive cardiomyopathy (HOCM) [21]. Moreover, the subsequent effect of this diminished LVOT gradient is evidenced by a statistically significant enhancement in the New York Heart Association (NYHA) functional class among patients following septal myectomy [22]. In the long term, this procedure demonstrates a substantial survival advantage, as postoperative patients exhibit survival rates comparable to those of the general population [23].

### 5.2. Alcohol Septal Ablation

For patients who are not deemed suitable surgical candidates or who choose not to undergo septal myectomy, alcohol septal ablation represents a viable alternative in the management of symptomatic hypertrophic obstructive cardiomyopathy (HOCM) and similarly possesses a Class I indication. Alcohol septal ablation (ASA) is an invasive procedure conducted by an interventional cardiologist wherein percutaneous access permits the operator to administer an alcohol-based solution directly to the septum. The targeted application of alcohol to the septal tissue reduces the septum’s thickness, with the intent of decreasing the left ventricular outflow tract (LVOT) gradient, which contributes to the downstream complications associated with HOCM. ASA has demonstrated a statistically significant improvement in LVOT gradient, New York Heart Association (NYHA) functional class, and mortality rates [24,25,26,27]. Of particular note, alcohol septal ablation has proven to be more effective in older patients, those with lower LVOT gradients, and those exhibiting reduced septal thickness [28]. While there has been extensive discussion regarding the comparison of outcomes between septal myectomy and alcohol septal ablation, the most notable difference is a higher incidence of long-term mortality in patients undergoing ASA compared to those who have undergone septal myectomy [29].

### 5.3. Radiofrequency Ablation

Radiofrequency ablation (RFA) represents an additional therapeutic option for hypertrophic obstructive cardiomyopathy (HOCM). Like other invasive interventions, RFA is primarily employed when symptoms remain unresponsive to first-line treatments. Furthermore, RFA may be indicated when alternative invasive methods are not practicable. This minimally invasive technique entails percutaneous access to position a radiofrequency electrode needle within the myocardial septum, allowing for the application of a current to ablate the hypertrophied septal tissue [30]. Research indicates that RFA can decrease the left ventricular outflow tract (LVOT) gradient, diminish septal thickness, and enhance the New York Heart Association (NYHA) functional class [30,31,32,33]. RFA is characterized by a low risk of complications, with one study reporting no incidence of bundle branch blocks or requirement for permanent pacemaker implantation post-operatively, which may be ascribed to the precision of the procedure [34].

### 5.4. High-Intensity Focused Ultrasound

An innovative, recently emerging technique involves utilizing the capabilities of ultrasound as a treatment modality rather than solely as a diagnostic option for cardiac diseases. Specifically, high-intensity focused ultrasound (HIFU) is proposed to deliver direct energy to cardiac tissue [35,36]. The direct energy administered via HIFU operates to ablate myocardial tissue, inducing necrotic changes in the myocardium through reduced coronary blood flow, thereby diminishing excess myocardial tissue. Although this modality is not presently approved for clinical use, preliminary trials demonstrate promise. This technique has been shown to directly reduce hypertrophied myocardial septal tissue in animal studies [36,37,38,39,40]. Further investigations have suggested that targeted contrast microbubbles can be injected to induce microlesions in myocardial tissue when combined with focused ultrasound, potentially reducing cardiomyocyte hypertrophy [41,42,43]. These contrast microbubbles contribute in various ways to assist with HIFU tissue ablation, enhancing image quality for precision targeting. More significantly, these microbubbles can directly travel to the target tissue, where applying HIFU energy induces their collapse. This high-energy microbubble collapse amplifies the thermal effects of HIFU ablation on myocardial tissue, further enhancing the reduction of hypertrophied septal tissue. Ongoing studies are evaluating the feasibility of HIFU for human treatment. This cutting-edge modality represents one of many emerging treatment techniques and offers a glimpse into the future direction of HOCM treatment.

### 5.5. Cardiac Device Implantation

At present, two categories of cardiac devices are conventionally utilized in patients with hypertrophic obstructive cardiomyopathy (HOCM). These include implantable cardioverter-defibrillators (ICDs) and cardiac resynchronization therapy (CRT) defibrillators. While both devices are employed in patients at elevated risk for sudden cardiac death (SCD) due to cardiac arrhythmias, CRT devices are primarily designated for symptomatic heart failure (HF) patients exhibiting a left bundle branch block (LBBB), with a left ventricular ejection fraction (LVEF) of less than or equal to 35%, and an increased QRS duration exceeding 150 ms [44]. The risk factors for SCD that may necessitate potential device implantation are succinctly summarized in Table 2. Limited literature has been published regarding CRT and hypertrophic cardiomyopathy (HCM), particularly since the release of the 2024 American Heart Association (AHA) guidelines. CRT possesses a Class 2a recommendation for patients with New York Heart Association (NYHA) Classes III–IV, presenting with an LVEF of less than 50% and LBBB. Although some instances have documented the advantages of CRT in HCM patients experiencing impaired left ventricular (LV) function and ventricular desynchronization, a study conducted by the Mayo Clinic in 2016 did not demonstrate a statistically significant difference in the improvement in LV systolic function or the composite endpoint of mortality, cardiac transplantation, or left ventricular assist device (LVAD) placement between HCM patients with an LVEF lower than 50% who received CRT treatment compared to those who underwent medical therapy alone [45]. Nonetheless, the presence of HCM in conjunction with ventricular dyssynchrony is insufficient to qualify a patient for CRT. Cardiologists contemplating CRT implantation should consider a QRS duration of at least 120 ms, as this parameter is independently correlated with all-cause mortality and heart failure hospitalizations [46]. Furthermore, CRT should be considered for patients who develop LBBB with a QRS duration greater than 150 ms following procedures such as percutaneous transluminal septal myocardial ablation or surgical myomectomy. The placement of an ICD is indicated as a Class 1 procedure in patients with a prior cardiac event (including SCD, ventricular fibrillation (VF), or sustained ventricular tachycardia (VT)). It holds a Class 2a recommendation for patients with a high five-year risk estimate who present with at least one of the following: a family history of SCD, significant left ventricular hypertrophy (LVH), unexplained syncope, apical aneurysm, or an LVEF less than 50%. Excluding the criteria above, a patient exhibiting late gadolinium enhancement (LGE) on cardiac magnetic resonance (CMR) or non-sustained ventricular tachycardia (NSVT) upon ambulatory monitoring would receive a Class 2b indication for ICD placement. Lastly, if none of the criteria are satisfied, the placement of an ICD is regarded as a Class 3 intervention and may incur potential risks.

Implantable cardioverter defibrillators (ICDs) can be introduced utilizing two distinct methods. Transvenous ICDs (TV-ICDs) are inserted through venous access, navigating through the tricuspid valve to be positioned within the right ventricle. Subcutaneous ICDs (S-ICDs), as indicated by their nomenclature, have the lead generator implanted subcutaneously, residing above the breastbone [47]. Effectively, S-ICDs assist in mitigating the risks associated with vascular access-related complications, the requirement for fluoroscopy during implantation, and the potential for lead dislodgement [48]. A multicenter study conducted in 2024 determined that S-ICDs were more frequently recommended to patients categorized as having a low to intermediate risk for sudden cardiac death (SCD) and found that the safety and efficacy of converting ventricular fibrillation rhythms to sinus rhythms were comparable between the S-ICD and TV-ICD groups, with no statistically significant differences observed in the incidence of adverse events and both appropriate and inappropriate shocks [49]. Furthermore, a meta-analysis performed by da Silva et al. revealed that individuals with hypertrophic cardiomyopathy (HCM) who received an S-ICD exhibited a markedly lower incidence of device-related complications, including migration and infection, compared with HCM patients who received a TV-ICD [50]. Consequently, these studies suggest that adoption of S-ICDs may expand, considering their favorable outcomes.

Simultaneously, it is crucial to acknowledge that there have been documented instances in the literature regarding the application of left ventricular assist devices (LVADs) for patients with end-stage heart failure (HF) secondary to hypertrophic cardiomyopathy (HCM). The evaluation for LVAD in patients classified under New York Heart Association (NYHA) Classes III–IV, who experience decompensation while awaiting cardiac transplantation, is associated with a Class 2a recommendation. The cases in question pertain to HCM patients who may not be viable candidates for medical therapy or implantation of defibrillators, as well as who may prove to be unsuitable candidates for surgical myomectomy or alcohol septal ablation [51]. While these cases illustrate the potential of LVAD as a bridging therapy to transplantation, LVAD patients with HCM may still present an elevated risk for complications, including thrombosis, stroke, infection, right heart failure, and aortic regurgitation [52]. This conclusion is substantiated by the research conducted by Niamat et al., which indicated that HCM patients undergoing LVAD exchange during their wait for heart transplantation faced significant risks, including not only exit-site infections, respiratory failure, and right-sided heart failure but also the necessity for device re-exchange and decreased survival six years following the exchange [53].

## 6. Gene Editing

**Figure 2:**
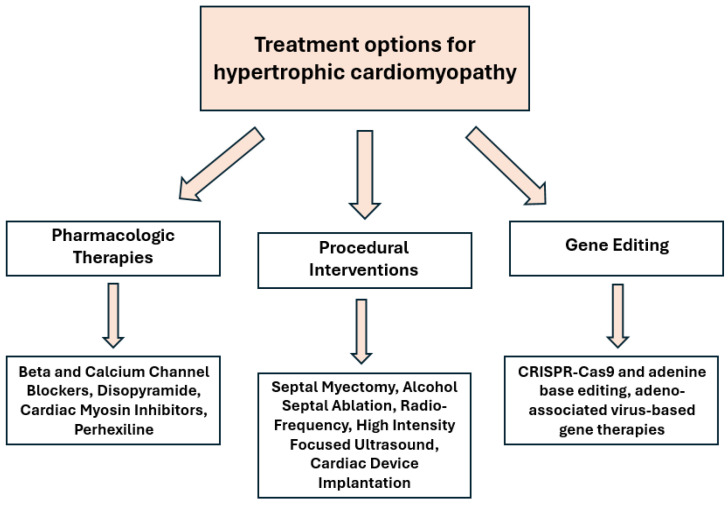
Types of therapies for HCM.

The traditional treatment for hypertrophic cardiomyopathy (HCM) primarily emphasizes the management of symptoms rather than addressing the underlying genetic etiology. Current interventions aimed at symptom reduction include the avoidance of strenuous physical activity, administration of beta blockers and non-dihydropyridine calcium channel blockers, septal myectomy, and, depending on the degree of disease progression, the use of implantable cardioverter-defibrillators to prevent sudden cardiac death. As depicted in Figure 2, these strategies represent the cornerstone of management for patients experiencing symptomatic HCM. However, recent advancements in gene therapy, particularly those related to genomic editing and replacement, have demonstrated potential in targeting the molecular mutations understood to be the pathological foundations of HCM. Researchers have identified two genetic mutations within the cardiomyocytes of patients with HCM—specifically, the sarcomeric genes MYH7 and MYBPC3—which are most frequently associated with the onset of the condition. The MYH7 sarcomeric gene encodes a beta-myosin heavy-chain protein, whereas the MYBPC3 gene is responsible for encoding myosin-binding protein C; both proteins are essential for the proper function and contractility of the heart [54]. These genes are critical for adequate cardiac function and represent significant targets for emerging developments in genetic therapy.

Over the past two decades, the application of adeno-associated virus serotype 9 (AAV9) as a viral vector for therapeutic gene delivery has demonstrated significant promise in addressing key genetic etiologies of various cardiomyopathies at their fundamental levels. Compared to alternative AAV variants, AAV9 has shown remarkable effectiveness in its approach to cardiomyocyte transduction. Its high specificity for these cells, facilitated primarily through incorporating a troponin T (Tnnt2) promoter, enables the maintenance of adequate and stable levels within the cardiomyocytes [55]. Furthermore, it is efficient for delivering other prominent genetic tools, such as CRISPR-Cas9 and adenine base editing (ABE). CRISPR-Cas9 comprises an RNA-guided endonuclease that cleaves double-stranded segments of targeted DNA. Conversely, adenine base editing (ABE) modifies DNA bases, converting adenine to guanine without inducing breaks in the DNA strand. Reichart et al. compared ABE and CRISPR-Cas9 in targeting the MYH7 R403Q mutation, a recognized pathogenic variant associated with hypertrophic cardiomyopathy (HCM). Their research indicated that administering a single dose of cardiac-specific ABE, particularly the ABE8e variant, through AAV9, restored function and structure in approximately 70% of ventricular cardiomyocytes in mouse models of HCM. This genomic editing approach effectively prevented muscle thickening and fibrosis within the targeted cardiomyocytes. The ABE8e variant was identified as both safer and more effective than CRISPR9, which succeeded in silencing the mutant gene but was associated with a limited therapeutic window, as higher dosages resulted in impaired contractile function [56].

Comparable research by Chai et al. investigated the utilization of AAV9-delivered ABE to rectify the MYH7 R403Q mutation. By meticulously examining various combinations of ABE and guide RNA (sgRNA), the researchers identified a specific combination that targeted the R403Q mutation in cardiomyocytes while effectively circumventing off-target genes [57]. This optimal combination, comprising ABEmax-VRQR and h403_sgRNA, was conjugated to AAV9 and initially evaluated on induced pluripotent stem cells (iPSCs) sourced from human subjects diagnosed with hypertrophic cardiomyopathy (HCM). Following base editing, these iPSCs were differentiated into cardiomyocytes, demonstrating normal function devoid of the characteristic mechanical or metabolic dysfunctions associated with HCM. More specifically, these cells exhibited restored normal ATP consumption and contractility. The ABEmax-VRQR-sgRNA-AAV9 combination underwent further testing in neonatal mice harboring either homozygous or heterozygous MYH7 R403Q mutations. The group with homozygous mutations exhibited extended survival, increasing from the average 7-day lifespan typically observed in this cohort to approximately 15 days, in the heterozygous mutation group, which more closely models the characteristic human disease, editing resulted in approximately 35% correction of the mutation in vivo, ultimately averting the onset of fibrosis, hypertrophy, and metabolic abnormalities in these cells [57]. These genetic methodologies, the targeted gene, and the key findings of the studies are summarized in Table 3.

An ongoing clinical trial conducted by the biotechnological company Tenaya Therapeutics is evaluating the efficacy of AAV9-based gene therapy in human patients. MyPEAK-1 (ClinicalTrials.gov identifier: NCT05836259) is a Phase 1b/2 dose-escalation and expansion study that assesses the safety (Phase 1b) and efficacy (Phase 2) of TN-201, an AAV9-based gene therapy aimed at the genetic treatment of hypertrophic cardiomyopathy (HCM) in participants with MYBPC3-associated HCM. The current model investigates two cohorts receiving varying doses of the vector genome. Cohort 1 comprises three participants receiving a dose of 3 × 10^13^ vector genomes per kilogram (vg/kg), while Cohort 2 consists of three participants receiving a higher dose of 6 × 10^13^ vg/kg. Ultimately, the researchers intend to expand the study to a maximum of 24 participants, who will be divided to receive either of these doses [58]. The AAV9-based gene therapy, TN-201, aims to deliver a normal MYBPC3 gene to cardiomyocytes via intravenous infusion, promoting increased MyBP-C3 protein levels. Recent data suggest improvements in transgene activity among patients in the lower-dose cohort, with one patient demonstrating a 3% increase in MyBP-C3 protein. The current priorities of the research team include completing long-term follow-ups and analyzing trends in the higher-dose cohorts. Based on the current observations, TN201 demonstrates a favorable safety profile, practical gene expression, and initial indications of clinical benefit [58].

## 7. Conclusions and Future Directions

Hypertrophic cardiomyopathy (HCM) has historically been an overlooked and underdiagnosed condition, characterized by limited treatment options and a generally poor prognosis. Recently, substantial advancements in diagnostic tools and pharmacologic interventions for individuals afflicted with HCM have redefined the approach to managing this disease. The emergence of artificial intelligence (AI) has paved the way for novel detection methods for HCM, as exemplified by studies such as a review conducted by Madaudo and colleagues, which examined the potential integration of ChatGPT (from GPT-3.5 to GPT-4) into cardiology practice while highlighting its advantages, including patient education and imaging interpretation, as well as its challenges, such as a lack of contextual understanding and the complexities of clinical decision making [59]. Additionally, another study by Desai and colleagues demonstrated that AI-powered electrocardiogram algorithms exhibit high sensitivity and specificity in detecting HCM, further illustrating the potential of AI to enhance cardiology practices [60]. With the introduction of first-in-class medications, such as cardiac myosin inhibitors, the promising outcomes associated with genetic editing, and the support of artificial intelligence in early detection, the future of HCM appears more optimistic than ever. However, notwithstanding the significant progress made in this field, HCM continues to be underdiagnosed. It is imperative to increase efforts to enhance awareness regarding the detection and early diagnosis of HCM, ensuring that timely intervention and treatment are initiated, ultimately leading to improved patient outcomes.

## Figures and Tables

**Table 1:** Proposed AHA/ACC guidelines for diagnosing HCM in adults and children [6].

Diagnostic Criteria for Hypertrophic Cardiomyopathy in Adults Versus Children	
**Adults**	Identification of a maximal end-diastolic wall thickness of ≥15 mm in any part of the left ventricle (with no other cause of hypertrophy in adults).
	An end-diastolic wall thickness ranging from 13 to 14 mm may serve as a diagnostic indicator, notably when correlated with a family history of hypertrophic cardiomyopathy (HCM) or a positive genetic test.
**Children**	The diagnostic criteria exhibit variability; a z-score exceeding 2.5, adjusted for body surface area, may be suitable for identifying early hypertrophic cardiomyopathy (HCM) in asymptomatic children without a family history. Conversely, a z-score greater than two may be employed for children with a familial history of HCM or those who have received a positive genetic test result.

**Table 2:** SCD clinical risk factors based on 2024 AHA/ACC guidelines [15]. HCM: hypertrophic cardiomyopathy; CMR: cardiac magnetic resonance; SCD: sudden cardiac death; LVOTO: left ventricular outflow tract obstruction; NSVT: non-sustained ventricular tachycardia; LGE: late gadolinium enhancement.

Sudden Cardiac Death Risk Factors in Adults with HCM	
Left Ventricular Hypertrophy	CMR or echocardiographic imaging demonstrating wall thickness > 30 mm in any segment.
Family History of SCD	First-degree relatives < 50 years of age who experienced SCD attributed to HCM.
Left Ventricular Apical Aneurysm	Apical aneurysm with transmural scar, regardless of size.
Unexplained Syncope	At least 1 episode of syncope that is deemed to be unrelated to vasovagal or LVOTO causes.
HCM in the Setting of Low Ejection Fraction	CMR or echocardiographic evidence of ejection fraction < 50%.
Non-Sustained Ventricular tachycardia upon Ambulatory Monitoring	NSVT defined as >3 beats at a rate of 120 beats per minute or higher occurring over 24 to 48 h of monitoring.
Late Gadolinium Enhancement on CMR	Fibrosis of >15% of the left ventricular mass detected by LGE.

**Table 3:** Comparison of different genetic approaches, including CRISPR-Cas9, ABE8e, and ABEmax-VRQR, and their effects on the target gene MYH7.

Gene Editing Approaches				
Approach	Target Gene	Delivery Method	Model Studied	Key Findings
CRISPR-Cas9 [56]	MYH7 (R403Q)	AAV9 with cardiac-specific promoter	Mouse models with HCM	The mutated MYH7 (R403Q) was successfully silenced, leading to a decrease in hypertrophy and fibrosis. However, due to a restricted therapeutic window, dosage increases negatively affected cardiac contractility.
ABE8e (Adenine Base Editing) [56]	MYH7 (R403Q)	AAV9 with Tnnt2 promoter	Mouse models with HCM	ABE8e reduced hypertrophy and fibrosis in cardiac muscle within cardiomyocytes possessing mutated MYH7 (R403Q) alleles and was deemed to be safer and more effective than CRISPR.
ABEmax-VRQR [57]	MYH7 (R403Q)	AAV9 with sgRNA h403_sgRNA	iPSC-derived human cardiomyocytes and neonatal mice	Cardiomyocytes exhibiting mutated MYH7 (R403Q) alleles, targeted by ABEmax-VRQR, demonstrated restored contractility and ATP metabolism. An approximately 35% correction in mutated alleles was noted in vivo for heterozygous mice, coupled with prolonged survival in models with homozygous mutations.

## Data Availability

Not applicable.
